# The diatomic molecular spectroscopy database

**DOI:** 10.1186/s13321-020-00433-8

**Published:** 2020-05-11

**Authors:** Xiangyue Liu, Stefan Truppe, Gerard Meijer, Jesús Pérez-Ríos

**Affiliations:** Fritz-Haber-Institut der Max-Planck-Gesellschaft, Faradayweg 4-6, 14195 Berlin, Germany

**Keywords:** Franck–Condon factors, Molecular spectroscopic constants, Open database, Laser cooling

## Abstract

**Motivation:**

The spectroscopy of diatomic molecules is an important research area in chemical physics due to its relevance in astrochemistry, combustion chemistry, and ultracold physics. However, there is currently no database where the user can easily retrieve, in a useful format, the spectroscopic constants of a given molecule. A similar situation appears concerning the vibrational Franck–Condon factors for diatomic molecules, a crucial parameter to infer laser cooling prospects for molecules. To address this problem, and inspired by the idea that data should be open and freely accessible, we have developed a user-friendly website (https://rios.mp.fhi.mpg.de) where the user can retrieve spectroscopic constants and Franck–Condon factors in useful formats.

**Implementation:**

In this database, the spectroscopic constants of the ground states and first excited states of the diatomic molecules are accessible from the website and can be retrieved in readable formats. The website is implemented within the LAMP web service stacks. In particular, using Linux as the operative system, Apache as the HTTP Server, MySQL as the database management system, and PHP as the programming language for the web. Furthermore, the user can register and upload new data. This project is licensed under the Free-Libre/Open Source Software (FLOSS) license Apache License 2.0 which allows free and open access to the codes as well as efficient collaboration in the maintenance of the software.

**Conclusions and impact:**

The present data-driven website presents essential information in a user-friendly manner and may help the chemical physics community to identify molecules that should be explored through spectroscopic techniques.

## Introduction

We are living in the so-called information revolution, in which information is faster and more accessible than ever. In this era, data needs to be easily accessible and fast shared among users. In addition, the data has to be shown in an interactive and friendly manner. Science, as a cornerstone of social development, needs to be up to date within that paradigm. In particular, the field of chemical physics is already evolving in that direction. Some useful databases have been released to the public through different websites such as HITRAN [1], ExoMol [2], NIST Chemistry WebBook [3], and OSDB [4], among others [2, 5, 6]. These sites focus on spectral properties of molecules relevant for astrophysics and atmospheric physics applications, nevertheless they do not contain data regarding the spectroscopic constants of diatomic molecules. Only the NIST Chemistry WebBook site shows the spectroscopy constants of diatomic molecules. However, in the NIST Chemistry WebBook, the data cannot be retrieved in convenient, readable formats such as XML, JavaScript Object Notation (JSON), or comma-separated values (CSV).

The importance of controlling the internal and external degrees of freedom of diatomic molecules is growing in chemical physics owing to their applications in quantum information [7], ultracold chemistry [8, 9], and the study of physics beyond the standard model [10, 11]. Most of these applications rely on laser cooling and trapping techniques, which have been achieved for a few molecules. These techniques are suitable for molecules showing almost vertical Franck–Condon factors (FCFs), which depend on the spectroscopic constants of the ground and excited electronic states. Thereby, developing a database containing the spectroscopic constants and FCFs, among the different states, will help to identify the best candidates for molecular laser cooling.

In this paper, we present a database linked to an interactive website devoted to the spectroscopic constants of polar diatomic molecules for the ground and first excited electronic states, as well as to the calculation of FCFs assuming a Morse potential shape for all the states involved. The database contains the spectroscopic constants of diatomic molecules taken from Huber and Herzberg [12], which is the most complete compendium of molecular spectroscopy for diatomic molecules. The website is interactive and allows the user to upload new data upon acceptance by the web managers. In that way, the database is dynamic and may experience a growth motivated by new spectroscopy measurements.

## Construction and content

**Fig. 1:**
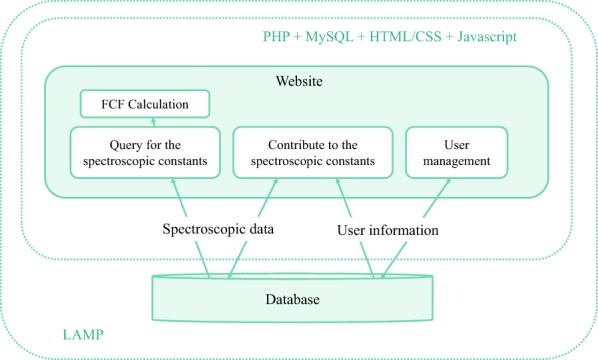
The infrastructure of the website

The website is implemented with Linux, Apache, MySQL, and PHP (LAMP) on the backend. Bootstrap [13] provides a convenient Hypertext Markup Language (HTML), Cascading Style Sheet (CSS) and JavaScript framework for developing responsive, mobile-first websites. Thus, the website can automatically resize and adapt the layout when loaded on a variety of devices with different screen sizes. The infrastructure of the website is shown in Fig. 1. The main functionalities of the website include querying and contributing to the spectroscopic data, as well as the calculation of the FCFs. PHP and MySQL implement data query and edition on the database and manage user information. The webpages are generated dynamically by PHP with HTML/CSS and JavaScript. In particular, JavaScript is used for fast calculation and visualization of the FCFs.

**Fig. 2:**
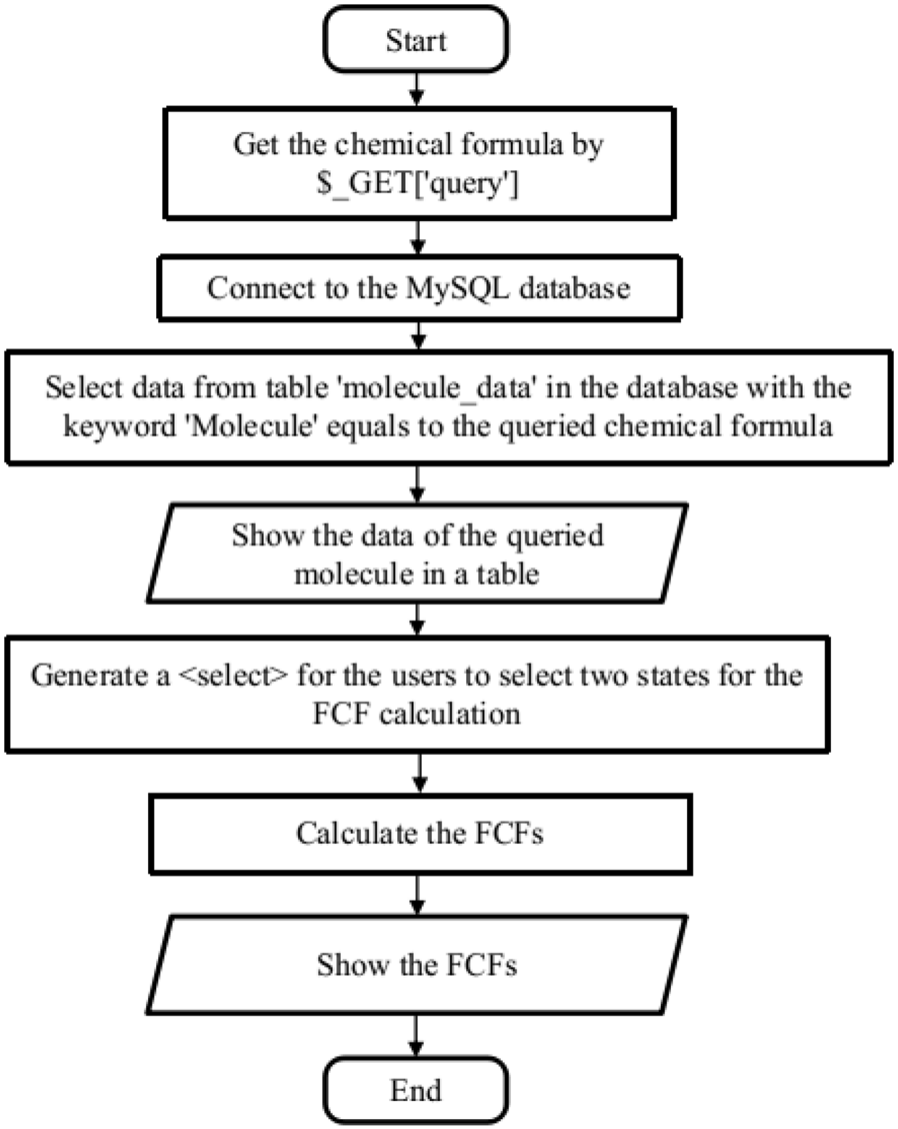
Flowchart of search_data.php

### Database construction

The spectroscopic constants of diatomic molecules are taken from Huber and Herzberg [12], and saved in a MySQL database. The molecules in the database and the states of all the molecules are indexed with non-null integer numbers “idMol” and “idAll_in” in table “molecule_data”, respectively, while the latter is used as the primary key of the table. Each state has a symbol represented in the Latex format, which is saved as a string in the database. The reduced mass of the molecule is also saved as a non-null float. The spectroscopic constants include the minimum electronic energy, $T_e$ (in cm$^{-1}$), the harmonic frequency $\omega _e$ (in cm$^{-1}$), the first anharmonic correction $\omega _e x_e$ (in cm$^{-1}$), the equilibrium rotational constant, $B_e$ (in cm$^{-1}$), the anharmonic correction to the rotational constant, $\alpha _e$ (in cm$^{-1}$), the centrifugal distortion constant, $D_e$ (in cm$^-1$), the binding energy, $D_0$ (in eV), the equilibrium internuclear distance $R_e$ (in Å), and the ionization potential, *IP* (in eV), for a given molecule in a given state. The allowed values of these constants are either float or “NULL”. The reference from which the data were taken, as well as the date of the reference are also recorded. The database also records the contribution information of each record, including the contributor, identified by “id_user”, and the date when the data are input into the database.

### Website functionality

The main function of the website is to allow users to search in the database for the spectroscopic constants of a given diatomic molecule. It is implemented in “search_data.php”, as shown in Fig. 2. The keyword for the query is the chemical formula of the molecule obtained by the HyperText Transfer Protocol (HTTP) GET method from the input field. Then a query is made in the MySQL table “molecule_data”, which retrieves rows containing spectroscopic information of the queried molecule. If the molecule exists in the database, the query results are shown in a table and are available in CSV format. Then an HTML <select> tag is generated according to the available electronic states of this molecule, allowing users to select two electronic states for the Franck–Condon factor calculation. The FCFs between the first several vibrational levels of the ground state and first electronically excited states are also calculated on the fly, which then can be used to make bar and density plots, as the ones shown in Fig. 6 with the aid of the JavaScript library D3.js [14].

**Fig. 3:**
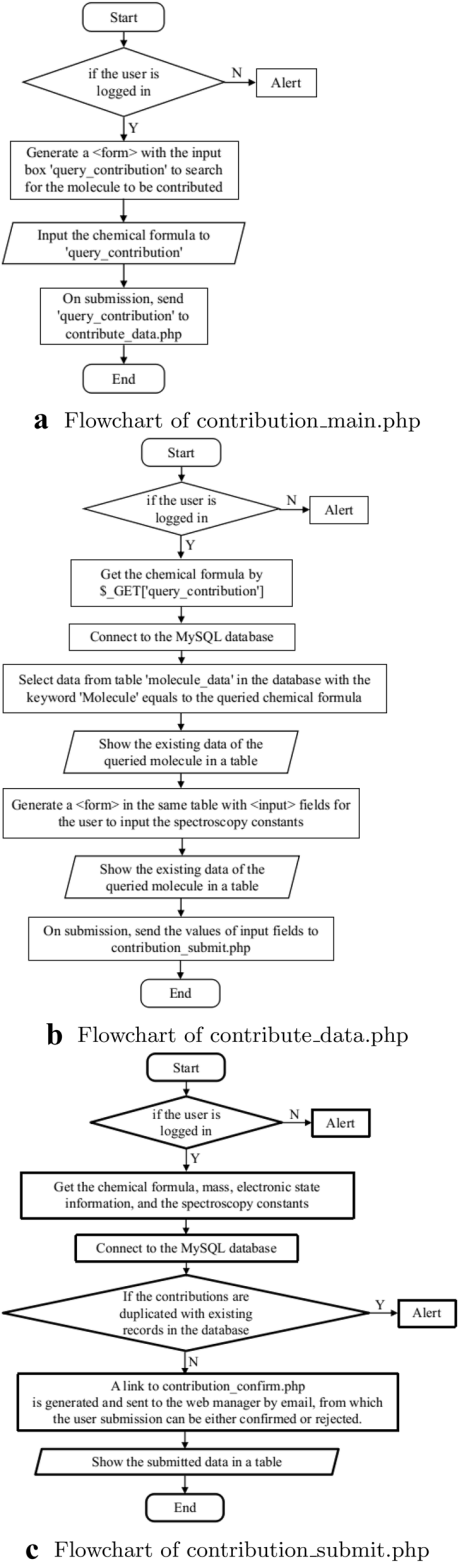
Flowcharts of the implementation on user contributions

**Fig. 4:**
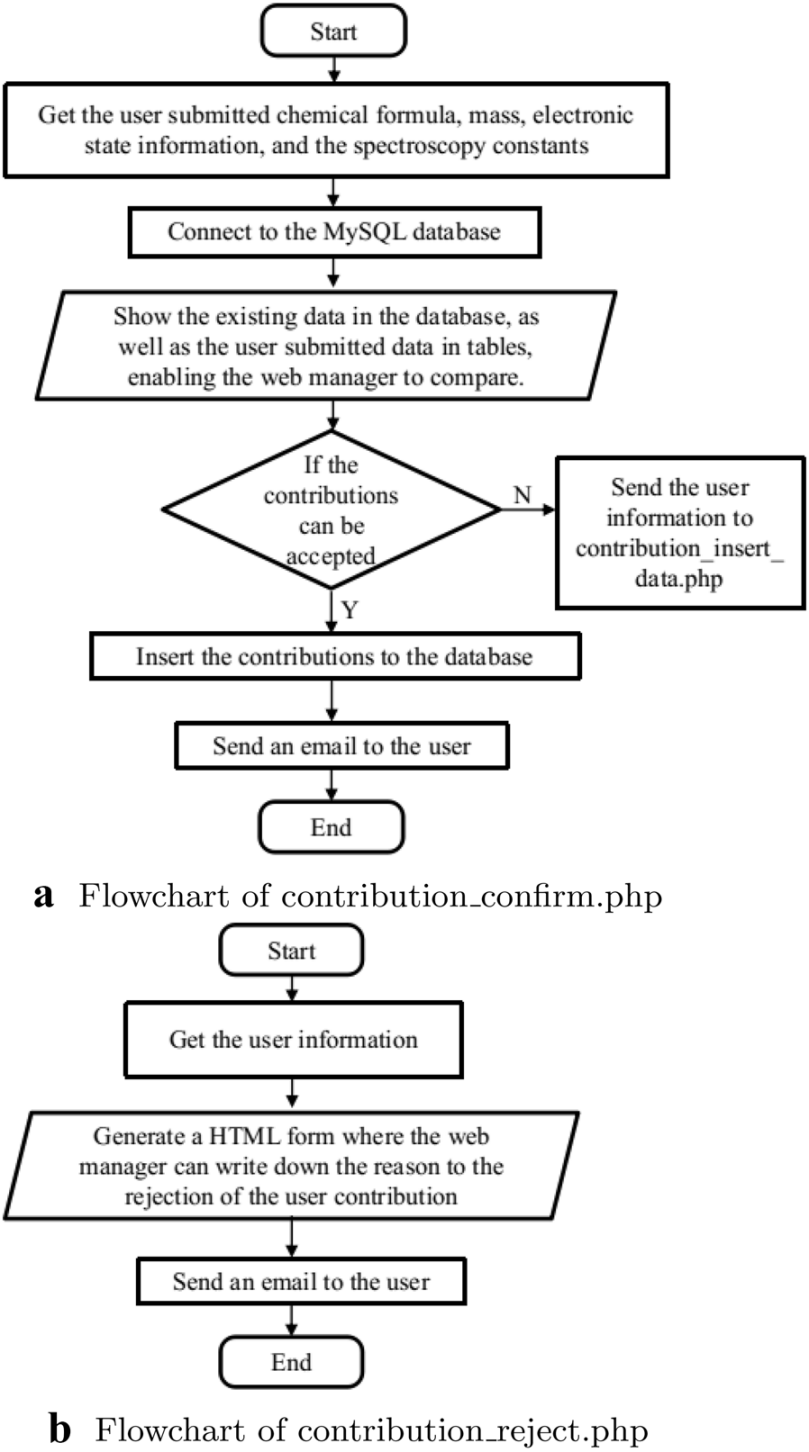
Flowcharts of the implementation on confirming or rejecting user contributions

The registered users can contribute to the spectroscopic data into the database via a web page interface containing input forms. This is implemented in “contribution_main.php”, “contribution_data.php”, “contribution_submit.php”, and the authorization of web manager is implemented in “contribution_confirm.php”, and “contribution_reject.php”. After the submission is confirmed, the data will be uploaded into the database by “contribution_insert_data.php”. The flowcharts are shown in Figs. 3 and  4. In “contribution_main.php”, if the users are logged in, an input field named “query_contribution” is shown for the users to enter the chemical formula of the molecule they want to contribute to the database. On submission, the action of the input field is “contribute_data.php”, which gets the chemical formula and retrieves data from the table “molecule_data” in the database with the queried chemical formula. The results appear in a table. Then an HTML <form> is generated in the same table for the users to enter the spectroscopic constants, with an action “contribution_submit.php” which processes the input data and make a duplicate check which compares if the spectroscopic constants in the current submission have the same values with the existing data in the database. The newly uploaded data is shown in a table to the user, while a link to “contribution_confirm.php” is generated and sent to the web managers by email. With this link, “contribution_confirm.php” shows the existing data in the database and the user submission in tables, so that the web managers can make a comparison and decide to either confirm or reject the contribution (Fig. 4a). The data provided by the user is checked in a two-step approach. First, it is checked if the journal referenced by the user is indexed in the Web of Science. Second, the data provided by the user is checked against the data in the paper. If the data is correct, then the contribution is accepted. In this way, owing to the review process it has passed, we ensure that the data provided by our website is of high quality. If the web managers decide to reject the contribution, “contribution_reject.php” will generate a HTML form for the web managers to write down the reasons for rejection (Fig. 4b), and send to the contributor by email.

### Franck–Condon factor calculations

The intensity of a vibronic transition between two states is proportional to the square of the overlap integral between the vibrational wavefunctions of these states. Therefore the FCFs indicate the favored vibrational transitions between different electronic states of a molecule. In this work, we assume that the interatomic potential is described by a Morse potential built from the spectroscopic constants from the database. This approximation is accurate for the description of the vibrational levels lying close to the minimum of the potential. For vibrational states lying nearby the minimum of the potential, the role of second and higher anharmonic corrections is negligible. Hence, a Morse potential is an adequate description of the potential.

The time-independent Schrödinger equation is solved numerically with the discrete variable representation (DVR) method [15, 16], which provides efficient and accurate numerical solutions to few-body quantum mechanical problems [17]. In DVR, the basis sets are associated with a set of quadrature points so that a diagonal matrix represents the potential matrix. In contrast, the kinetic energy operator contains non-diagonal terms. In this implementation, the number of the DVR quadrature points is set to 200 for the calculation of the vibrational wavefunctions leading to an error of less than 0.1$\%$. The overlap between the vibrational wavefunctions is calculated numerically using the trapezoidal rule.

## Utility and discussion

### Search in the database

**Fig. 5:**
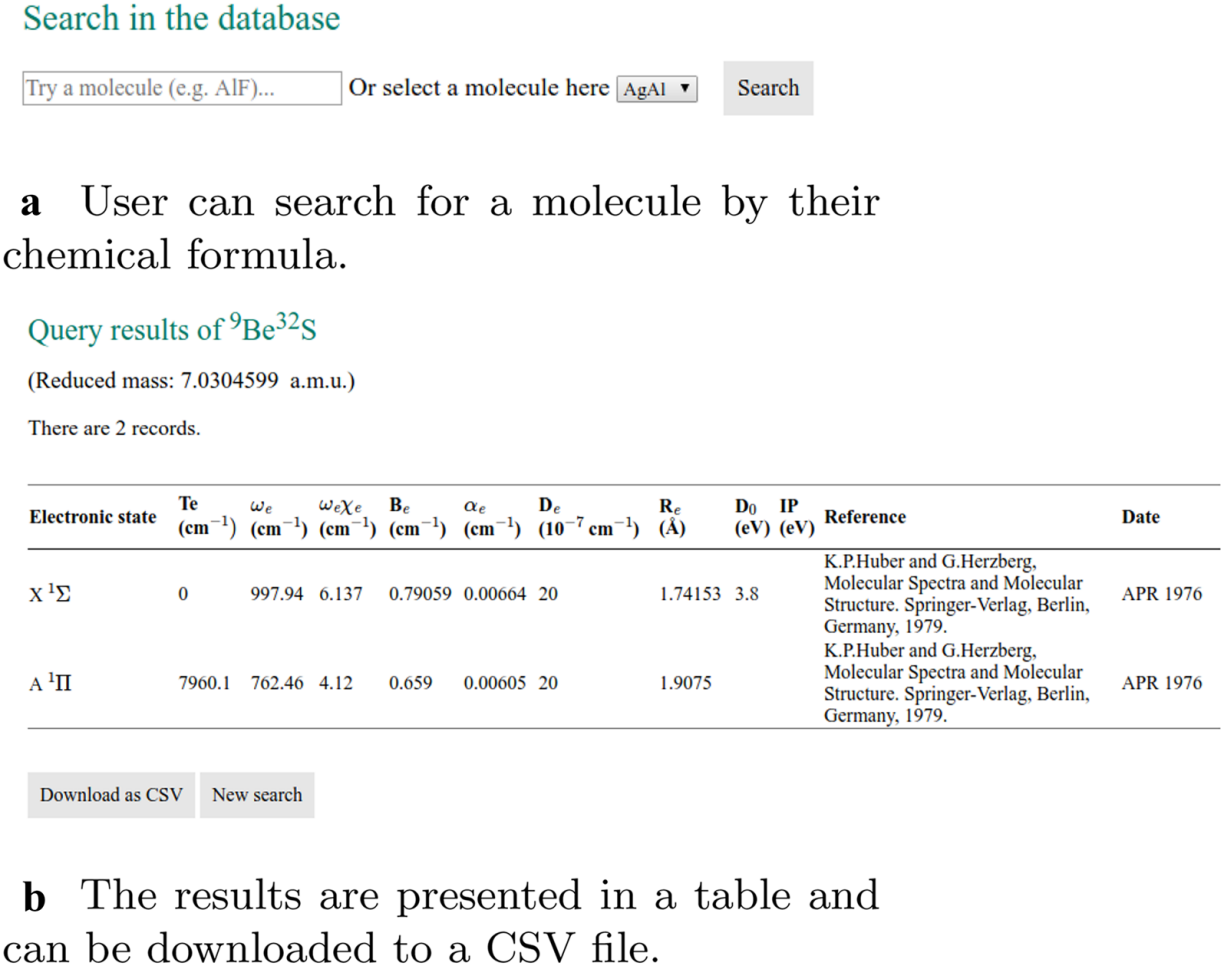
Search in the database

**Fig. 6:**
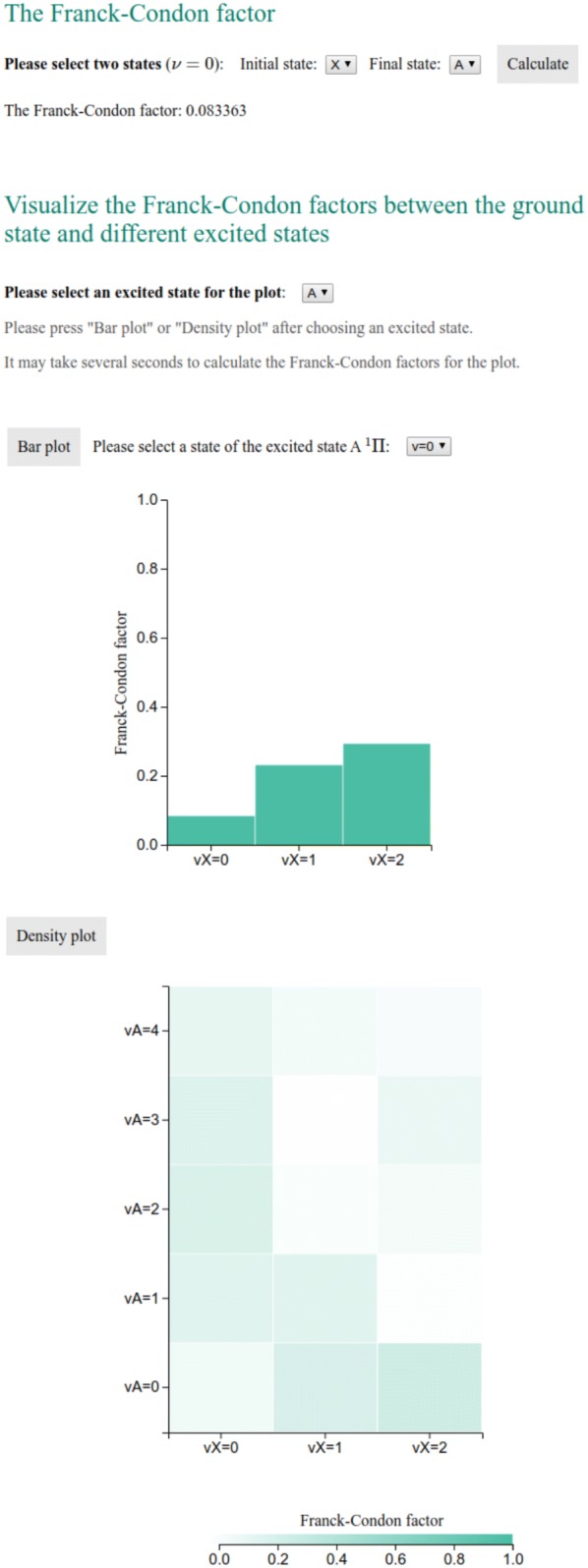
The Franck–Condon factor

The user can retrieve the spectroscopic constants from the database by searching by the chemical formula of molecules. The results are shown in a table and are available in CSV format (Fig. 5), which is convenient for processing. The FCFs of selected states for the molecules are calculated from the spectroscopic constants of the ground and excited states. As shown in Fig. 6, the FCFs between different vibrational states of the ground electronic state and the first excited electronic states can be shown in a bar plot or a density plot.

**Fig. 7:**
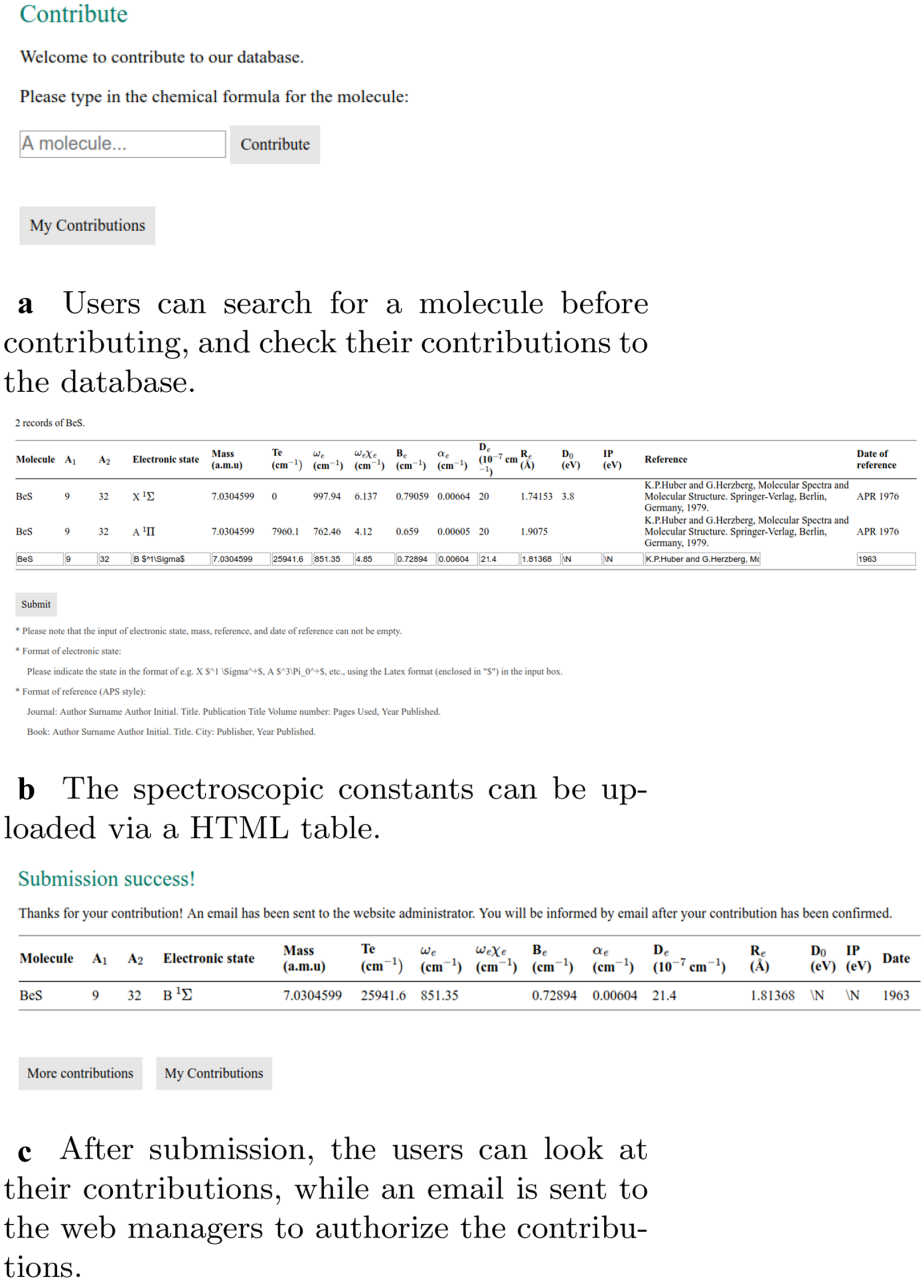
Contribute to the database

### Contribute to the database

**Fig. 8:**
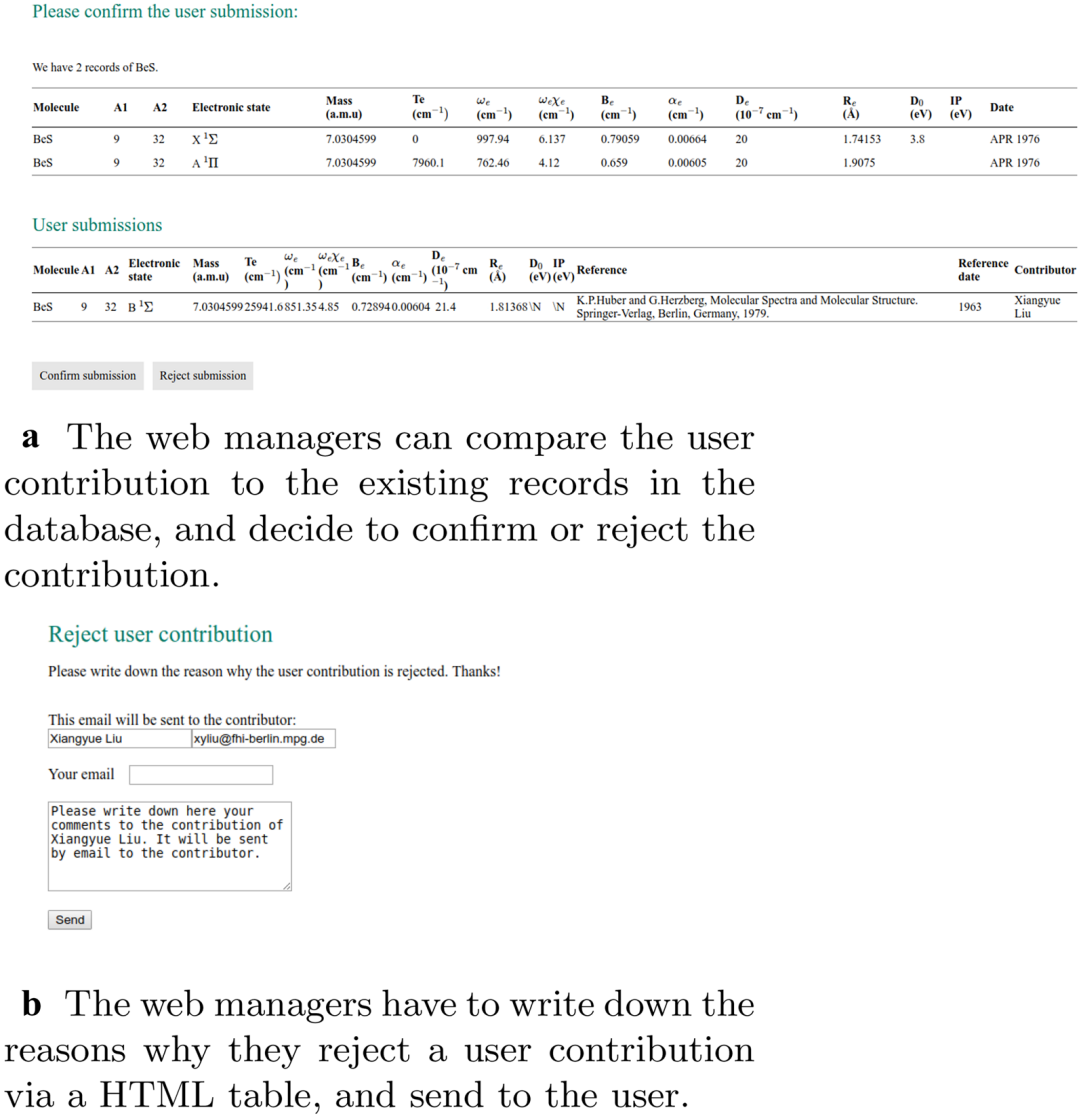
Authorization of user contributions by the web managers

After an initial search for a given molecule to know the present spectroscopic information, new information can be uploaded by the user to the database, as shown in panel (a) of Fig. 7. The spectroscopic information can be uploaded via an HTML form, while the reduced mass, and the reference information should be a non-null field. The electronic states must be uploaded in Latex format, e.g., X $^1\Sigma ^+$. The contributions are inserted into the database upon authorization of web managers (Fig. 8a), or if rejected by the manager, the reason will be sent to the contributor via email (Fig. 8b). Finally, users can also check their contributions to the database.

**Fig. 9:**
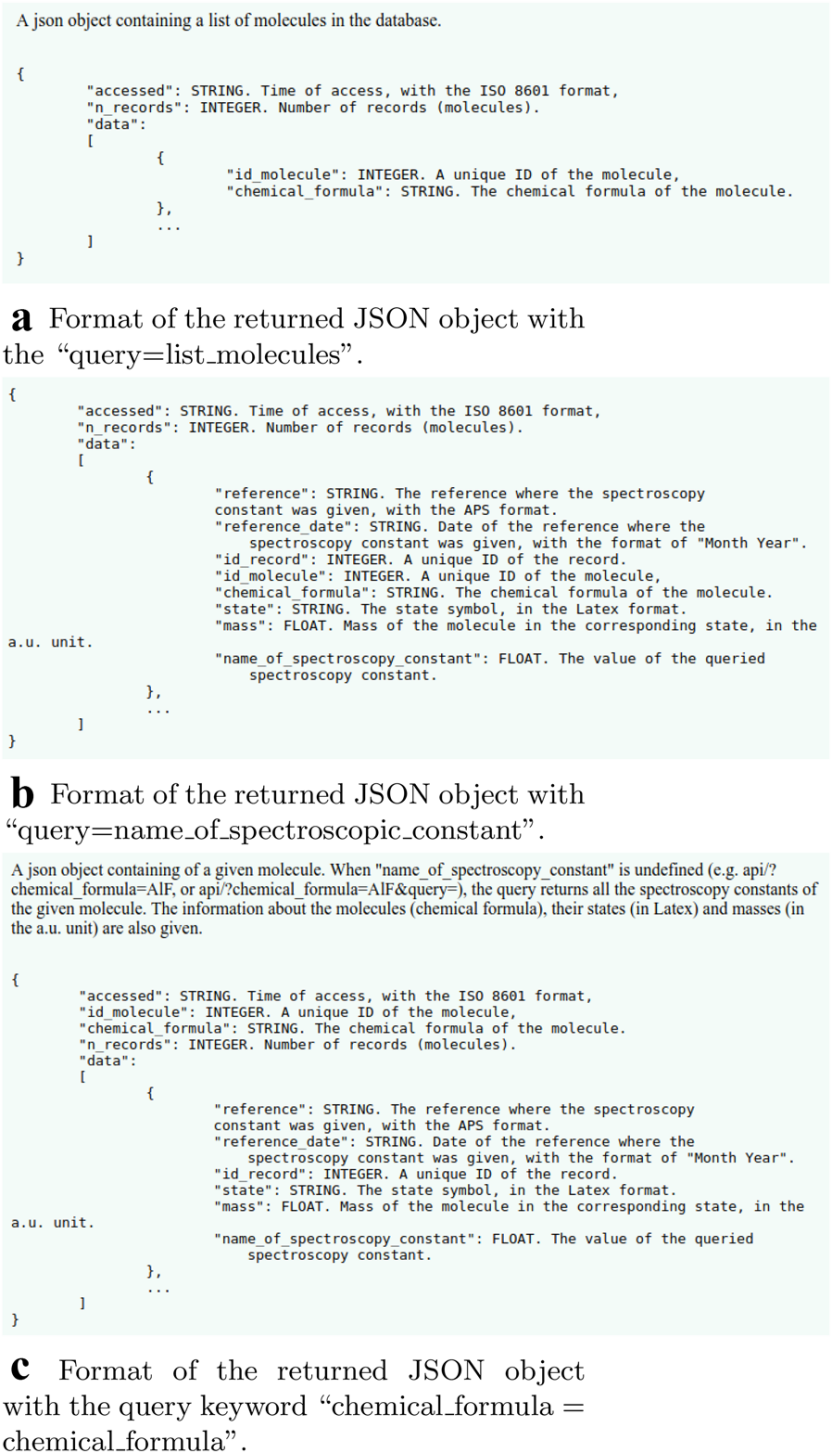
Sample returns of API’s

### The application programming interface (API)

An application programming interface (API) is provided for the users to query the spectroscopic information of the molecules in the database. The API returns JSON objects with the format shown in Fig. 9. With “/api/?query=list_molecules” a list of all the molecules in the database can be provided, which contains an array of objects where the molecules are indexed by “id_molecule” and labeled by their chemical formula. “/api/?query=name_of _spectroscopic_constant” returns spectroscopic constants of the ground and excited states of all the molecules having the information of this constant in the database. The detailed information about a molecule can be obtained by searching with “/api/?chemical_formula=chemical _formula&query=name_of_spectroscopic_constant”. For example, “api/?chemical _formula=AlF&query=Be”, returns a JSON object containing $B_e$ of AlF. When “name_of_spectroscopic_constant” is undefined (e.g. “api/?chemical_formula=AlF”, or “api/?chemical_formula=AlF&query=”), the query returns all the spectroscopic constants of the given molecule. The information about the molecules (chemical formula), their states (in Latex) and masses (in a.u.) are also given.

## Conclusions

The diatomic molecule spectroscopic database provides free access to the ground state and excited state spectroscopic constants of polar diatomic molecules, as well as FCFs between different electronic states. The database is managed dynamically by allowing the registered users to upload the spectroscopic data into the database. Currently (2020-04-11), we have only a small percentage of all the possible diatomic polar molecules throughout the periodic table. In particular, we count 130 molecules with a $\Sigma$ ground state, 34 molecules with a $\Pi$ ground state, 5 molecules with a $\Delta$ ground state, and no molecule with a $\Phi$ ground state. In future, we plan to upload more spectroscopic constants such as dipole moment and new functionalities to reach a more broad audience.

## Data Availability

The complete database is accessible through the API in the website https://rios.mp.fhi.mpg.de. The source code is available at https://github.com/hlslxy/DMSD under the Apache License.
